# The grief experience during the COVID-19 pandemic across different cultures

**DOI:** 10.1186/s12991-022-00397-z

**Published:** 2022-06-14

**Authors:** Frances Adiukwu, Leila Kamalzadeh, Mariana Pinto da Costa, Ramdas Ransing, Renato de Filippis, Victor Pereira-Sanchez, Amine Larnaout, Jairo M. Gonzalez-Diaz, Mario Eid, Zulvia Syarif, Laura Orsolini, Rodrigo Ramalho, Ramyadarshni Vadivel, Mohammadreza Shalbafan

**Affiliations:** 1 Department of Mental Health, University of Port Harcourt, Rivers Port harcourt, Nigeria; 2 Mental Health Research Center, Psychosocial Health Research Institute, Department of Psychiatry, School of Medicine, Iran University of Medical Sciences, Tehran, Iran; 3 Institute of Psychiatry, Psychology & Neuroscience, King’s College London, London, UK; 4 Institute of Biomedical Sciences Abel Salazar, University of Porto, Porto, Portugal; 5 South London and Maudsley NHS Foundation Trust, London, UK; 6 Department of Psychiatry, BKL Walawalkar Rural Medical College, Ratnagiri, 415606 Maharashtra India; 7 Psychiatry Unit, Department of Health Sciences, University Magna Graecia of Catanzaro, 88100 Catanzaro, Italy; 8 Department of Psychiatry, Columbia University, New York, NY USA; 9 Department of Child and Adolescent Psychiatry, New York University Grossman School of Medicine, New York, NY USA; 10 Psychiatry Department D, Faculty of Medicine of Tunis, Tunis El Manar University, Tunis, Tunisia; 11 UR Center for Mental Health - CERSAME, School of Medicine and Health Sciences, Universidad del Rosario, Bogotá DC, Colombia; 12 Clínica Nuestra Señora de La Paz, Bogotá DC, Colombia; 13 Barcelona Clínic Schizophrenia Unit, Neurosciences Institute, Hospital Clínic, Universitat de Barcelona, Barcelona, Spain; 14 Faculty of Medical Sciences, Lebanese University, Hadath, Lebanon; 15 Tarakan General Hospital, Jakarta, Indonesia; 16 Unit of Clinical Psychiatry, Department of Neurosciences/DIMSC, School of Medicine, Polytechnic University of Marche, Ancona, Italy; 17 Department of Social and Community Health, School of Population Health, The University of Auckland, Auckland, New Zealand; 18 Waikato District Health Board, Hamilton, New Zealand

**Keywords:** Grief, COVID-19, Culture

## Abstract

Grief is the physical or mental suffering experienced after a major loss, usually the death of a loved one. It is a universal experience, but sociocultural factors, such as cultural or ethnic identity and religious beliefs predict and shape the expression of grief. The circumstances under which people are experiencing grief during the coronavirus outbreak have adversely affected the grieving process. Unexpected deaths, social distancing rules and visitor restrictions in healthcare facilities have posed a heavier burden on the loss and have heightened the risk of grievers experiencing complicated or persistent grief. This concern led us, as early career psychiatrists (ECPs) from 14 different countries connected by the Early Career Psychiatrists Section of the World Psychiatric Association (WPA), to share our country-specific experiences on the mourning, grief tradition, and burial rites during the COVID-19 pandemic. In this paper, we discuss our experiences, similarities and differences with relation to the: ‘Effect of the pandemic on mourning’, ‘Restrictions and Guideline on burial rites due to the pandemic’, ‘Effect of the pandemic on social support’ and ‘Role of media and telecommunication on mourning practices and burial rites’. We conclude that while telecommunication means have attempted to bridge the gap and provide some form of social connectedness, the total and global effect of the pandemic is yet to be fully seen and understood.

## Background

Grief is the physical or mental suffering experienced after a major loss, usually the death of a loved one. The uncomplicated grieving process often encompasses physical distress, sadness, anxiety, confusion, intense longing for the deceased, rumination about the past, and fear about the future. Grief may also take the form of regret for something lost, remorse for something done, or sorrow for a mishap to oneself [1].

Grief is a universal experience, but sociocultural factors, such as cultural or ethnic identity and religious beliefs predict and shape the expression of grief [2]. Traditional funeral and burial rites offer a venue for the culturally and psychologically appropriate expression of loss-related emotions. Simultaneously, they provide a starting point for recovery and social cohesion [3].

## Main text

At the time of writing this publication, the deaths of more than 5 million people in the world from coronavirus disease 2019 (COVID-19) have left behind many millions bereaved [4]. Even those who have not lost someone directly to COVID-19 are currently susceptible to other types of loss, ranging from social or physical support to financial security to a loss of autonomy and safety [5]. The circumstances under which people are experiencing grief in the coronavirus outbreak have adversely affected the grieving process [6]. Unexpected deaths, social distancing rules and visitor restrictions in healthcare facilities have posed a heavier burden on the loss and have heightened the risk of grievers experiencing complicated or persistent grief [7]. Many governments have introduced policy measures and guidelines on funeral and burial rites to reduce the spread of COVID-19. However, concerns have been raised that being prevented from conducting appropriate funeral and burial rituals might hinder COVID-19 grievers from obtaining awareness of and adjusting to the reality of the death, and can eliminate an important occasion of social support [8]. This concern led us, as early career psychiatrists (ECPs) connected by the Early Career Psychiatrists Section of the World Psychiatric Association (WPA) [9, 10], to share our country-specific experiences on the mourning, grief tradition, and burial rites during the COVID-19 pandemic. We hoped that this collective endeavor providing perspectives from different World Health Organization (WHO) regions in countries severely affected by COVID-19 would strengthen mental health care policies to support families of isolated or deceased COVID-19 patients.

The funeral customs and cultural death-related rituals of some of the countries before and during the COVID-19 pandemic will be discussed in detail. We further review the guidelines on burial rites introduced by the governments during the COVID-19 pandemic, the impact of the COVID-19 pandemic on social support towards survivors and families of the deceased, and the effect of the media on mourning, burial rites, and rituals during the pandemic (Table 1). Lastly, we discuss the role of telecommunication in the mourning and burial rites during the pandemic.

**Table 1:** Grief and mourning practices before and during the COVID-19 pandemic across different countries

Country	Importance of grief tradition, mourning and burial rituals	Prohibition of rituals and practices	Restrictions of rituals and practices	Presence/absence of immediate family at burial of Covid-19-related death	Presence of guidelines for only Covid-19-related deaths	Decreased social support to families of the deceased
Italy	Yes	Yes	Yes	Absent	Yes	Yes
Paraguay	Yes	No	Yes	Absent	No	Yes
Nigeria	Yes	No	Yes	Present	No	Yes
Tunisia	Yes	No	No	Absent	Yes	Yes
United States of America	Yes	No	Yes	Present	No	Yes
Indonesia	Yes	Yes	Yes	Present	Yes	Yes
Lebanon	Yes	No	Yes	Present	No	Yes
New Zealand	Yes	No	Yes	Present	No	N/a
Colombia	Yes	Yes	Yes	Absent	No	No
Portugal	Yes	Yes	Yes	Absent	Yes	No
United Kingdom	Yes	Yes	Yes	Absent	Yes	No
India	Yes	Yes	Yes	Present	Yes	Yes
Iran	Yes	Yes	Yes	Present	Yes	Yes

### Grief and mourning practices

Grief and mourning practices follow religious and cultural processes, intertwined and with each having a significant impact on the community. Christian religion (particularly the Catholic faith) is the religion mainly practiced in Italy, Colombia, Paraguay and Portugal. Iran, Tunisia and Lebanon are predominantly Muslim countries, while Nigeria, the United States of America, Indonesia, India and New Zealand are multi religious. In all countries, mourning practices are an important aspect of the burial rites, usually involving a large gathering of family and friends (in Paraguay, Nigeria, Lebanon, Iran, Italy and native New Zealanders family denotes both nuclear and extended families) and lasting up to 2 weeks (around 7 days in Italy and 2 weeks in New Zealand). The process requires a lot of physical contact with each other as well as with the deceased person (the body is often washed and dressed by family members in Italy and Nigeria), sharing of food (large banquets are held by the Guajira, indigenous Colombians) and a procession of the dead person’ relatives through the community. The burial is done according to the religion of the deceased and afterwards, especially if the deceased is elderly, there is a celebration of life.

#### Effect of the pandemic on mourning

During the peak of the first wave of the pandemic, in almost all countries traditional rituals to honor the dead and comfort mourners were abbreviated or even completely banned. Some countries allowed events with a limited number of attendees (e.g., in Nigeria only 50 people were allowed to attend funerals for a country that usually has as much as 500 people at funerals); although most countries banned all mass gatherings, regardless of the number of participants. Often families were prohibited from coming into contact with the deceased and local authorities took the responsibility to bury them. In some countries like USA and Italy COVID-19 deaths warranted cremation, while in others, such as Colombia and India, burial in mass graves, whereas in Muslim cultures, both cremation and collective graves are strictly prohibited even at times of pandemic. Many religious rituals were also strongly affected. For instance, in Italy, during the first wave (early 2020) the last rites normally given to Catholics were not allowed or restricted to a few close relatives. In Tunisia, Islamic rituals of the body preparation and holding requiem for the dead were prohibited for people who died of COVID-19.

#### Restrictions and guideline on burial rites due to the pandemic

Early in the pandemic, most countries adopted guidelines and strict restrictions on funerals and memorial services for those who have lost their lives to COVID-19 and, in some countries, all deaths occurring during the pandemic, e.g. Paraguay. In Lebanon, no guidelines were created by the government and decisions in churches/mosques to limit funerals varied between regions. On the other hand, the USA, Indonesian, Indian, and Italian Government adopted the highest level of precautions given to their exceptional number of deaths. However, in some countries, such as Colombia, India and Iran, the COVID-19 measures were often violated in the early stages of the outbreak and especially by those in rural areas. In some cases, these restrictions contradicted the country’s religious practices (e.g., burying corpses in coffins in Indonesia, which is contrary to Islamic rituals).

#### Effect of the pandemic on social support

Social support has substantially decreased in most of the affected countries with social distancing rules, adding a further burden to the pain, and giving rise to the conditions of ‘complicated’ grief, in which loved ones have found themselves unable to overcome the trauma of loss. Physical distances and travel limitations have made it difficult for extended family and friends to reunite and support the immediate relatives of the deceased. This is especially the case in countries like Lebanon, Iran, Paraguay and India where funerals usually involve the nuclear and extended family members as well as neighbors, and even the whole village. Unfortunately, in many countries affected by the disease, the government and health institutes did not provide adequate social and psychological support. In countries like Tunisia and Iran many families who lost one of their members of COVID-19, experienced stigma instead of support due to the lack of adequate education and information provided by governments to people about the disease.

#### Role of media and telecommunication on mourning practices and burial rites

Social media and telecommunication has had a great impact in reducing the distances imposed by physical distancing during all phases of lockdown, and this also applies to religious and prayer rites in nearly all countries. Virtual funerals are an evolving resource for providing a sense of social connectedness and serve as an alternative to traditional grieving practices in the context of the COVID-19 pandemic. Furthermore, media plays a major role in informing the public about the guidelines of burial rites. However, it seems that the fear of COVID-19, the saturation of bad news and the repeated reports of the number of deaths had a distressing effect on people and impacted the ceremonies by families with deceased in many countries such as USA, India, Indonesia, and Iran.

### Complicated grief and mental health

Grief following the COVID-19 pandemic is estimated to resemble that which follows natural disasters [11]. It is expected that prolonged or complicated grief would be increased during the pandemic. This increase is not only as a result of the unprecedented number of confirmed COVID-19 cases and related deaths, but also due to social isolation, disrupted mourning practices and grieving processes experienced globally. Abnormal grief could cause significant impairment in the lives of individuals and could lead to psychopathologies such as major depressive disorder and anxiety disorder as well as poor quality of life [12].

## Conclusions

The COVID-19 pandemic affected the grieving process with respect to burial rites, mourning rituals and social support. While telecommunication means have attempted to bridge the gap and provide some form of social connectedness, the total and global effect of the pandemic is yet to be fully seen and understood.

## Data Availability

Not applicable.

## Abbreviations

COVID-19: Coronavirus disease 2019
ECPs: Early Career Psychiatrists
WPA: World Psychiatric Association
WHO: World Health Organization
